# The use of machine learning models for subdural hematoma detection: a single-arm meta-analysis

**DOI:** 10.1007/s10143-026-04422-7

**Published:** 2026-08-06

**Authors:** Suraj R. Dumasia, Warda Ahmed, Michelle Edavettal, Mackenzie Castellanos, Om H. Gandhi, Giuseppe Lanzino, Omar Choudhri

**Affiliations:** 1 Department of Neurosurgery, University of Pennsylvania, Philadelphia, PA USA; 2 Aga Khan University Medical College, Karachi, Pakistan; 3 Department of Neurosurgery, Mayo Clinic, Rochester, MN USA; 4 Department of Radiology, University of Pennsylvania, 15th Floor South Pavilion, Room 15-146 , 3400 Civic Center Boulevard, Philadelphia, PA USA; 5 Department of Neurological Surgery, University of Chicago, Chicago, IL USA

**Keywords:** Deep learning, Subdural hematoma, Machine learning, Artificial intelligence

## Abstract

Manual evaluation of non-contrast CT scans (NCTS) for detecting subdural hematoma (SDH) is time consuming, potentially inaccurate, and subjective to the expert analyzing them. In recent years, two deep learning (DL) algorithms have been popularly studied in this respect, namely convolutional neural networks (CNN) and U-Net architectures, the latter being a specialized type of CNN. We performed the first meta-analysis comparing various DL models for SDH detection. MEDLINE, Cochrane, Scopus, and Embase databases were searched from inception through December 2025. Studies evaluating ML model performance on an independent test dataset were included. The main outcome measures were sensitivity, specificity, diagnostic odds ratio (DOR), accuracy, and precision of CNN, U-Net, and hybrid DL models. Univariate meta-regression analyses were performed. 30 testing datasets incorporating 67,266 NCTS were included. U-Net demonstrated significantly higher sensitivity (0.916;*p* = 0.04) and precision (0.983;*p* = 0.001) while high specificity, DOR, and accuracy values were consistently observed across all DL techniques. Internal testing (*p* = 0.05) was a borderline significant predictor of high specificity while recent publication year (*p* < 0.001), U-Net architecture (*p* = 0.035), and 3D models (*p* = 0.022) emerged as significant moderators of high precision. The U-Net architecture was also a borderline significant predictor of high DOR (*p* = 0.049). While this single arm meta-analysis depicts potential superiority of U-Net models with respect to sensitivity and precision, these findings are based off only 4 pooled U-Net datasets in comparison to the 22 pooled for CNN architectures. Future well-powered studies evaluating the U-Net model are necessary to ensure a fair comparison of U-Net architectures to other DL designs before reaching to any definitive conclusions in this respect.

**Supplementary Information:**

The online version contains supplementary material available at 10.1007/s10143-026-04422-7.

## Introduction

Subdural hematomas (SDH) occur at a rate of 58.6 cases per 100,000 individuals annually in the United States with a predicted 78.3% increase in its burden by 2040 [1]. Falls and road traffic accidents constitute the most common causes of SDH development [2]. SDH typically presents as a crescent shaped blood collection between the dura and arachnoid mater, resulting from rapid acceleration/deceleration forces that disrupt the bridging veins traversing the subdural space [3].

Non-contrast computed tomography scans (NCTS) are the most commonly employed first line diagnostic modality for detecting SDH. Given the 41% mortality rate among moderate-severe SDH patients, accurate and timely diagnosis is critical [4]. Acute SDH (< 2 days) comprises a blood clot visualized as a hyperdense concavity crossing the suture lines and amounting to 50–60 Hounsfield Units (HU) on NCTS [5]. As protein degradation occurs, structural composition of the hematoma changes, rendering the region hypodense and isodense in the subsequent subacute (3 days – 3 weeks) and chronic (> 3 weeks) stages, respectively; this makes it challenging to accurately segment the hematoma from the normal brain parenchyma [6]. Moreover, falls and road traffic accidents, constituting the majority of SDH cases, also result in concomitant head injuries, such as subarachnoid hemorrhage and contusion related bleeds, in nearly 90% of patients [7]. This, together with the low quality of NCTS led to the exclusion of 36% of CT scans in a study of SDH patients suffering from head trauma [7]. Furthermore, manually analyzing biomedical images is both inefficient and ill-equipped at accurately extracting all relevant data, in addition to the results being subjective to the expert analyzing them. As such, detection of pathologies like SDH relies on the availability of experienced physicians, something particularly lacking at odd hours of the day [8].

In recent years, the utility of artificial intelligence (AI), particularly machine learning (ML), has been increasingly explored across healthcare domains [9]. Deep learning (DL), a subset of ML, has been extensively investigated for SDH detection with DL models primarily spanning convolutional neural networks (CNN) and U-Net architectures [10–12]. Other ML models such as support vector machines, random forests, and gradient boosting have been less frequently explored. To date, no meta-analysis has compared different ML models in SDH detection. While a meta-analysis of SDH detection utilizing DL models was conducted for studies published until 2022, this review failed to compare different DL designs [13]. In addition, many more studies exploring ML in this context have been published since then. Given that U-Net architectures recover lost spatial resolution during up-sampling, they are significantly different from CNN and hence, it is important to pool them separately.

To this end, this single-arm meta-analysis aimed to bridge the gap in existing literature by pooling sensitivity, specificity, accuracy, and precision values comparing all the different ML models for detecting SDH.

## Materials and methods

This meta-analysis was registered with PROSPERO (ID: CRD420251241389) and conducted in accordance with the Preferred Reporting Items for Systematic reviews and Meta-Analyses (PRISMA) guidelines [14].

### Search strategy

We performed a comprehensive search of the MEDLINE, Cochrane Central Register of Controlled Trials (CENTRAL), Scopus, and Embase to identify all relevant studies published from inception through December 2025 using keywords related to “artificial intelligence”, “machine learning”, “deep learning”, and “subdural hematoma”. Additional studies were identified by manual searching and snowballing by assessing the references of narrative reviews, systematic reviews, and meta-analyses done on similar and related topics. No time or language restrictions were set and the detailed search string for each database is stated in Supplementary Table 1.

### Study selection criteria

Studies were included if they met the following criteria:

1. Included adults > 18 years of age.
2. Evaluated an ML model.
3. Included a testing dataset for evaluating performance of the pertinent ML model.
4. Assessed segmentation of SDH from NCTS specifically.
5. Reported data for at least one of the stated outcomes of interest in a manner that could be analyzed.

Meanwhile, studies not meeting the inclusion criteria, including those assessing other non-ML AI models, were excluded from the analysis.

### Screening process and data extraction

All articles retrieved from the search strategy were imported into the Rayyan software. Duplicates were removed and two reviewers (WA and MC) independently screened the titles and abstracts of the initially retrieved articles. Subsequently, the full text of each shortlisted article was reviewed to assess whether it met the predefined selection criteria. Any discrepancies were resolved by consensus or by consulting a third reviewer (OC). If data were missing from the manuscript or the supplementary files, corresponding authors were contacted to gather additional data.

### Extracted parameters

Data was extracted by two authors (WA and ME) independently with any discrepancies being resolved by consensus or by a third reviewer (OC). The parameters extracted included sensitivity, specificity, diagnostic odds ratio (DOR), accuracy, and precision of SDH segmentation by different ML models. The primary outcome of this study was to assess how different DL architectures differ across the abovementioned parameters. The secondary outcome of this meta-analysis was to evaluate the same for other non-DL ML models. The definitions of the extracted parameters were standard and are defined in detail in Supplementary Table 2. A meta-analysis of any outcome was performed provided that at least two studies reported it.

### Quality assessment

The Quality Assessment of Diagnostic Accuracy Studies-2 (QUADAS-2) tool was used to assess the quality of studies included in our systematic review [15]. Publication bias was assessed for any meta-analysis which included at least ten studies. Funnel plots were used to detect publication bias with the Egger’s regression intercept and two-tailed p value quantifying the visual asymmetry observed in cases of significant publication bias (*p* < 0.05).

### Statistical analysis

All meta-analyses were single arm meta-analyses performed using Comprehensive Meta-Analysis Version 4. The pooled proportions for each outcome of interest were reported with forest plots used to visually represent each meta-analyzed outcome. The random effects model was utilized for pooling all outcomes of interest. Subgroup analyses were performed on the basis of the specific DL architecture used (CNN, U-Net architecture, hybrid deep learning models).

Heterogeneity was assessed using the Higgins I^2^ statistic, where I^2^ > 50% was considered significant heterogeneity. In case of significant heterogeneity, a sensitivity analysis was performed by removing one test dataset at a time. Salehinejad et al. tested a model designed majorly using deep CNNs with boosting algorithms used to refine the values attained [16]. Therefore, while the study was pooled in the CNN subgroup, a sensitivity analysis was also performed by removing both the internal and external testing models by Salehinejad et al. to note whether this affected the results. Additionally, in case of significant publication bias being noted, a sensitivity analysis excluding all testing datasets with small sample sizes was performed. A small sample size was defined as one falling in the lower 25% of all sample sizes pooled for that outcome. Furthermore, a univariable meta-regression analysis was performed in case of significant heterogeneity provided that at least ten studies had been meta-analyzed. The covariates used for building the regression models included year of publication, sample size of the test set, AI model used, type of testing (internal, external), and dimensions of the AI model used (2D, 3D, 2D and 3D). A sensitivity meta-regression analysis was performed for all covariates, removing one test dataset at a time and assessing the impact of doing so on the association observed; results for this were only reported in case of any alterations being noted. For all regression analyses, the omnibus Q value alongside the residual heterogeneity was stated. A p-value of < 0.05 was considered significant for all analyses.

## Results

The initial literature search yielded 1006 articles of which 22 were finally included in this meta-analysis [10–12, 16–34] (Supplementary Fig. 1).

## Study characteristics

A total of 67,266 NCTS were included in this meta-analysis. Of the 30 test datasets included, 22 studied CNN models, 4 studied the U-Net model, and 4 assessed hybrid DL algorithms. 54.8% of included models were tested on internal datasets. Of those utilizing internal testing, the majority were test samples held-out from the training dataset (*n* = 11, 73.3%) while the rest were test samples independent of the training dataset. No study involving non-DL ML models was found eligible for inclusion. The detailed study characteristics are outlined in Table 1. Most included studies had low risk of bias (Supplementary Fig. 2).

**Table 1:** Characteristics of included studies

Author	Year	Country	Specific Type of DL	Training	Training Data Source	Testing Type	Testing Dataset	Test Sample Size	Stage of SDH
Qin et al.[10]	2024	China	CNN - DeepLabV3 + CNN	Yes	Single Center	1 internal	Blinded set independent of training set	70	All
Ahmed et al. [11]	2024	Iraq	U-Net - CSR-UNet	Yes	Single Center	1 internal	Internal hold-out set	46	aSDH
Kaya et al.[12]	2023	Turkey	CNN - Mask R-CNN	Yes	Single Center	1 internal*	Internal hold-out set and the public CQ500 dataset	511	aSDH
Hassan et al.[17]	2023	USA	CNN - Viz Ai	Pretrained	NA	1 external	Patients enrolled in the EMBOLISE trial	230	subacute and cSDH
Colasurdo et al.[34]	2023	USA	CNN - Viz Ai	Pretrained	NA	1 external	Retrospectively enrolled patients at a single center	263	All
Chilamkurthy et al.[33]	2018	India	2D CNN	Yes	Multicentered across 20 sites	1 internal, 1 external	Internal hold-out set and external CQ500 dataset	21,095 and 491	aSDH and cSDH
Lee et al.[32]	2019	USA	2D CNN	Yes	Single Center	2 internal	Two blinded retrospective and prospective datasets independent of training set	200 and 196	aSDH
Salehinejad et al.[16]	2021	USA	2D CNN^#^	Yes	Single Center	1 internal, 1 external	Internal hold-out set and external single center data	3518 and 5965	Not specified
Seyam et al.[31]	2022	Switzerland	3D CNN - AIdoc Medical	Pretrained	NA	1 external	Prospectively enrolled patients at a single center	4450	All three
Wang et al.[30]	2021	China	2D CNN + RNN	Yes	2019-RSNA dataset	1 internal, 2 external	Internal 2019-RSNA dataset; External CQ500 and PhysioNet-ICH datasets	2214, 491 and 75	aSDH
Ye et al.[29]	2019	China	3D CNN + RNN	Yes	Multicentered across 3 sites	1 internal	Internal hold-out set	194	Not specified
Zhou et al.[28]	2022	China	2D CNN ResNet-18 and DenseNet-121	Yes	Single Center	2 internal	Two internal hold-out sets	280 and 280	Not specified
Danilov et al.[27]	2020	Russia	2D CNN - ResNeXt	Pretrained	NA	1 external	Patients enrolled at a single center	401	Not specified
Neves et al.[26]	2023	USA	CNN - Caire ICH vR1	Pretrained	NA	1 external	Retrospectively enrolled patients at a single center	510	Not specified
Kang et al.[25]	2023	South Korea	2D U-Net	Yes	RSNA and AI Hub	1 external	Patients from a tertiary hospital in Korea and the open Qure.ai dataset	537	Not specified
Rava et al.[24]	2021	USA	CNN - Canon’s ^AUTO^Stroke Solution ICH	Pretrained	NA	1 external	Retrospectively enrolled patients at a single center	302	Not specified
Fasla et al.[23]	2025	Sri Lanka	2D CNN	Yes	RSNA dataset	1 internal, 1 external	Internal hold-out dataset and external RSNA dataset	225 and 150	All
Ahmed et al.[22]	2025	Iraq	2D UNet	Yes	Physionet dataset	1 internal	Internal hold-out dataset	71	Not specified
D’ Angelo et al.[21]	2024	Germany	3D U-Net - Siemens Healthineers (Forchheim, Germany)	Pretrained	NA	1 external	Retrospectively enrolled patients at a single center	502	aSDH
Gibson et al.[20]	2022	India, Germany, Canada, USA	Hybrid 3D/2D CNN	Yes	Multicentered across 7 sites	1 internal, 1 external	Internal hold out set from 7 internal centers; External data from 3 external centers	2832 and 16,764	aSDH and subacute SDH
Roshan et al.[19]	2024	USA	Hybrid 3D/2D CNN – Viz.ai	No	Pretrained	1 external	Retrospectively enrolled patients at a single center	4203	All
Huang et al.[18]	2025	Taiwan	3D CNN	Yes	Single center	1 internal, 1 external^$^	Internal hold out set; External CQ500 dataset	200	aSDH

*CNN* convolutional neural networks, *DL* deep learning, *USA* United States of America, *aSDH* acute subdural hematoma, *cSDH* chronic subdural hematoma, *RSNA* Radiological Society of North America, *NA* not applicable, *ResNeXt* Residual Networks with External Transformations, *ICH* intracranial hemorrhage, *AI* artificial intelligence, *RNN* recurrent neural networks, *EMBOLISE* A Study of the Embolization of the Middle Meningeal Artery With ONYX™ Liquid Embolic System In the Treatment of Subacute and Chronic Subdural Hematoma; CSR, Convolution squeeze excitation residual module; *Mixed internal-external, but predominantly internal; ^#^Gradient boosting used for refinement; ^$^Only external test results used since internal set SDH specific sample size was not available

### Outcomes of interest

A) Sensitivity

**Fig. 1:**
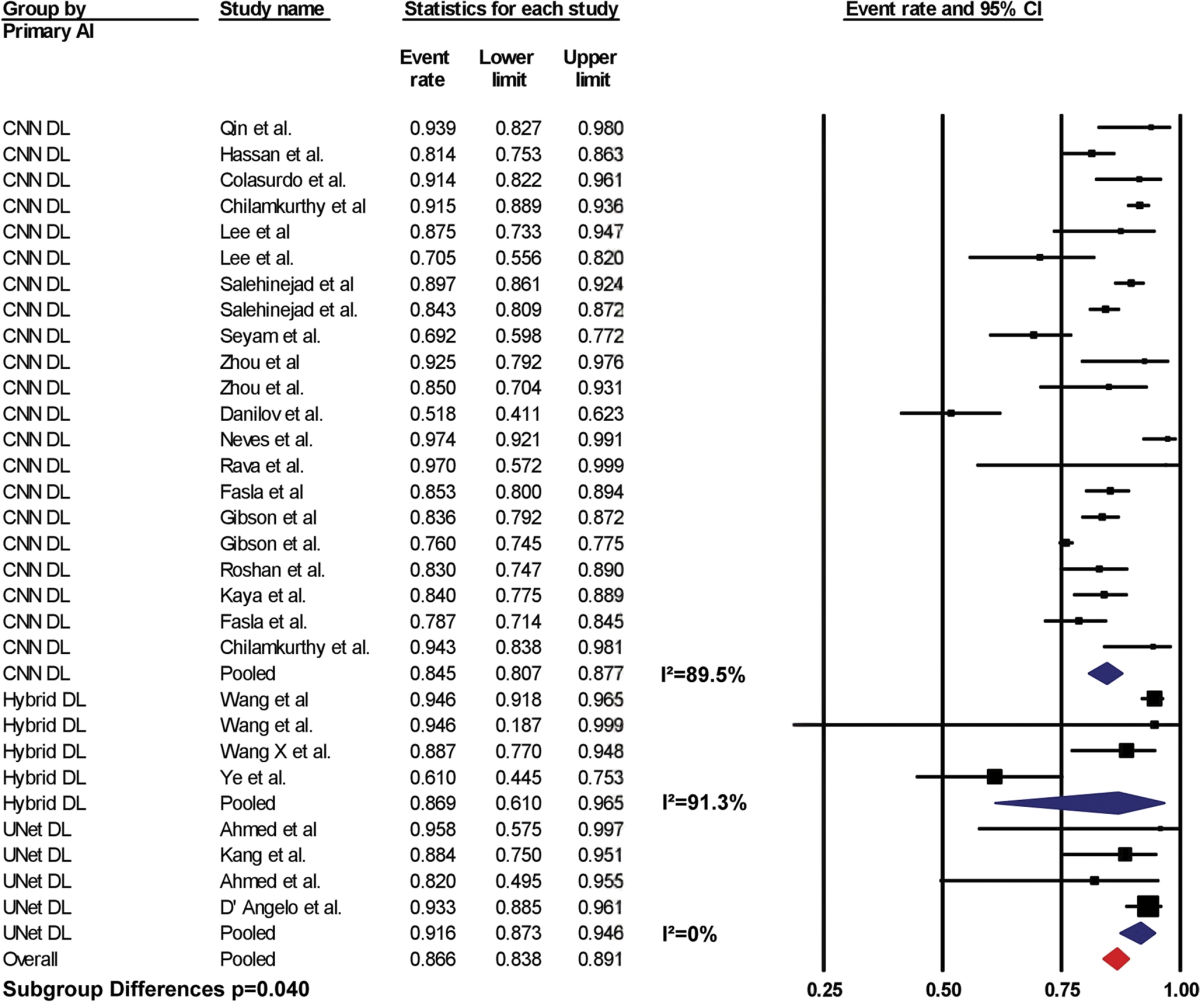
Forest plot depicting pooled sensitivity values for CNN DL, Hybrid DL, and U-Net DL architectures. Legend: AI, artificial intelligence; CI, confidence interval; DL, deep learning; CNN, convolutional neural networks

This outcome was reported by 19 studies, yielding 29 point estimates overall. Among DL models, the pooled sensitivity of CNN, U-Net, and hybrid models was 0.845 (95% CI [0.807, 0.877]; I^2^ = 89.5%; *n* = 21), 0.916 (95% CI [0.873, 0.946]; I^2^ = 0%; *n* = 4), and 0.869 (95% CI [0.610, 0.965]; I^2^ = 91.3%; *n* = 4), respectively (Fig. 1). Significant subgroup differences were observed (*p* = 0.040). Removing the study by Ye et al. significantly reduced heterogeneity in the subgroup of hybrid DL models (0.931 (95% CI [0.884, 0.960]; I^2^ = 25.3%) [29].

None of the five assessed covariates was significantly associated with the logit event rate in the random-effects meta-regression models (Supplementary Fig. 3A). Significant publication bias was observed (Egger’s intercept: 2.06; *p* = 0.007) (Supplementary Fig. 3B). After excluding small testing datasets, the results remained unchanged with significant subgroup differences (*p* = 0.008) still favoring U-Net (0.920 (95% CI [0.870, 0.952]; *n* = 2) and hybrid DL (0.927 (95% CI [0.855, 0.965]; *n* = 2) models over CNN (0.838 (95% CI [0.796, 0.873]; *n* = 17) [11, 22, 24, 28–30, 32].

B) Specificity

**Fig. 2:**
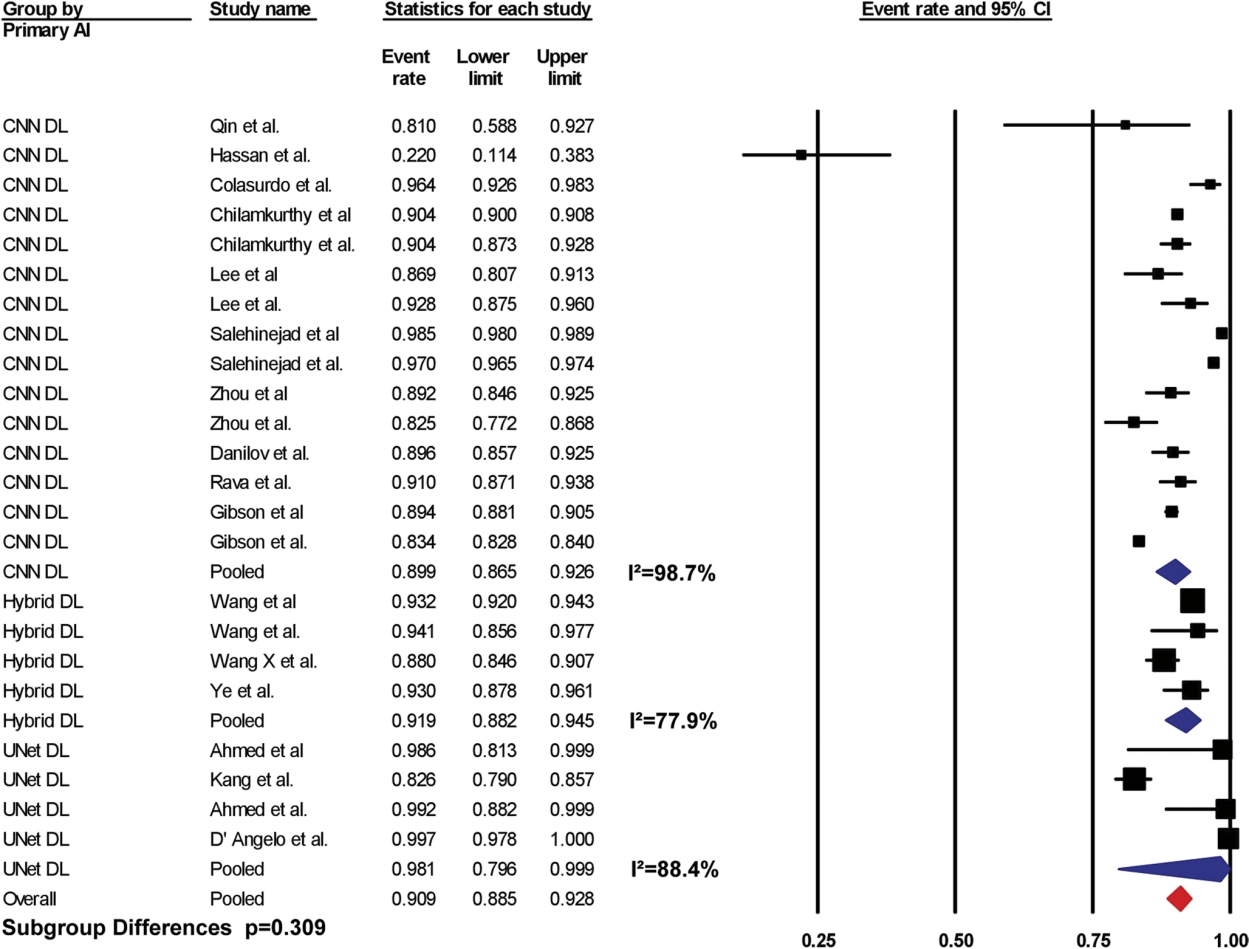
Forest plot depicting pooled specificity values for CNN DL, Hybrid DL, and U-Net DL architectures. Legend: AI, artificial intelligence; CI, confidence interval; DL, deep learning; CNN, convolutional neural networks

This outcome was reported by 15 studies, generating 23 point estimates overall. Among DL models, the pooled specificity of CNN, U-Net, and hybrid models was 0.899 (95% CI [0.865, 0.926]; I^2^ = 98.7%; *n* = 15), 0.981 (95% CI [0.796, 0.999]; I^2^ = 88.4%; *n* = 4), and 0.919 (95% CI [0.882, 0.945]; I^2^ = 77.9%; *n* = 4), respectively (Fig. 2). No significant subgroup differences were observed (*p* = 0.309). On removing the external CQ500 test model by Wang et al., there was a significant reduction in the heterogeneity observed in the subgroup of hybrid DL models (0.932 (95% CI [0.920, 0.942]; I^2^ = 0%) with subgroup differences becoming significant (*p* = 0.045) [30].

The overall heterogeneity was 97.9%. On performing meta-regression analysis, type of testing (Q = 3.78, df = 1, *p* = 0.05, residual I^2^ = 96.3%) and model dimensions used (Q = 9.39, df = 3, *p* = 0.028, residual I^2^ = 96.88%) emerged as significant predictors of specificity (Supplementary Fig. 4 A). Internal testing (β = 0.553, *p* = 0.05) possessed borderline significance in predicting higher specificity while hybrid 3D/2D models (β = −0.898, *p* = 0.028) were associated with significantly lower specificity. No publication bias was observed (Egger’s intercept: 2.35; *p* = 0.219) (Supplementary Fig. 4B).

C) Diagnostic Odds Ratio (DOR)

**Fig. 3:**
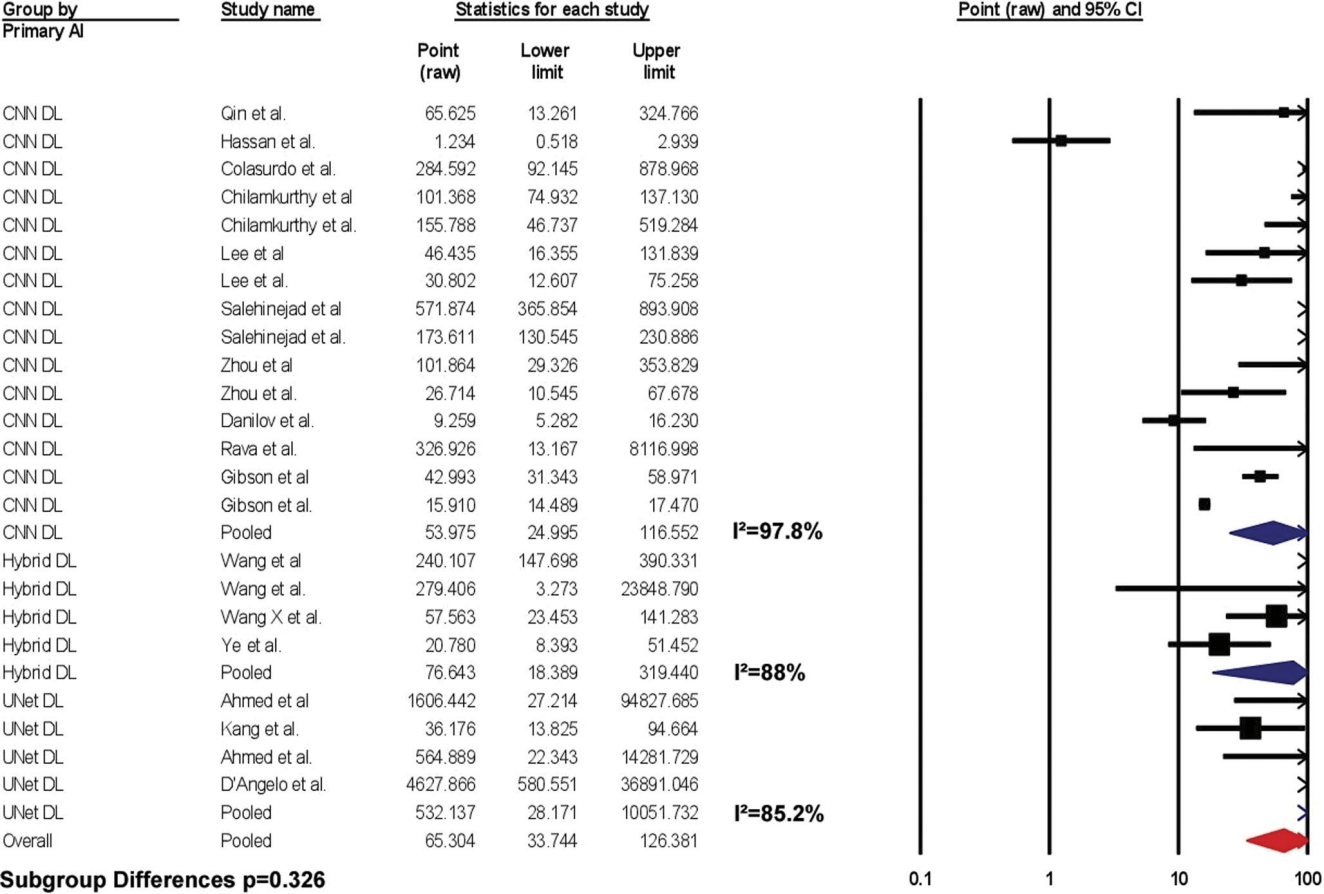
Forest plot depicting pooled diagnostic odds ratios for CNN DL, Hybrid DL, and U-Net DL architectures. Legend: AI, artificial intelligence; CI, confidence interval; DL, deep learning; CNN, convolutional neural networks

Combining sensitivity and specificity, DOR was calculated for 15 studies, generating 23 point estimates overall. Among DL models, the pooled DOR of CNN, U-Net, and hybrid models was 54 (95% CI [25, 116.6]; I^2^ = 97.8%; *n* = 15), 532.1 (95% CI [28.2, 10051.7]; I^2^ = 85.2%; *n* = 4), and 76.6 (95% CI [18.4, 319.4]; I^2^ = 88%; *n* = 4), respectively (Fig. 3). No significant subgroup differences were observed (*p* = 0.326). On removing the study by Kang et al., heterogeneity reduced to 0% in the U-Net subgroup (2336.5 (95% CI [469.2, 11634.5]; I^2^ = 0%) with significant subgroup differences being observed (*p* < 0.001). On removing the study by Wang et al., there was a significant reduction in heterogeneity observed in the subgroup of hybrid DL models (37.8 (95% CI [15, 95.5]; I^2^ = 39%); subgroup differences remained insignificant (*p* = 0.238).

The overall heterogeneity was 97.1%. On performing meta-regression analysis, primary AI (Q = 3.87, df = 2, *p* = 0.145, residual I^2^ = 97.1%) and model dimensions used (Q = 6.04, df = 3, *p* = 0.110, residual I^2^ = 93.5%) did not emerge as significant predictors of DOR (Supplementary Fig. 5A). U-Net models (β = 2.02, *p* = 0.049) possessed borderline significance in predicting a higher DOR while hybrid 3D/2D models (β = −1.40, *p* = 0.035) were associated with significantly lower DOR. Significant publication bias was observed (Egger’s intercept: 3.26; *p* = 0.033) (Supplementary Fig. 5B). After excluding small testing datasets, CNN (55.8 (95% CI [24.2, 128.6]; *n* = 13), U-Net (373.7 (95% CI [3.23, 43232.1]; *n* = 2), and hybrid DL (123.8 (95% CI [30.7, 500]; *n* = 2) models persisted in depicting comparable DORs (*p* = 0.497) [10, 11, 22, 29, 30, 32].

D) Accuracy

**Fig. 4:**
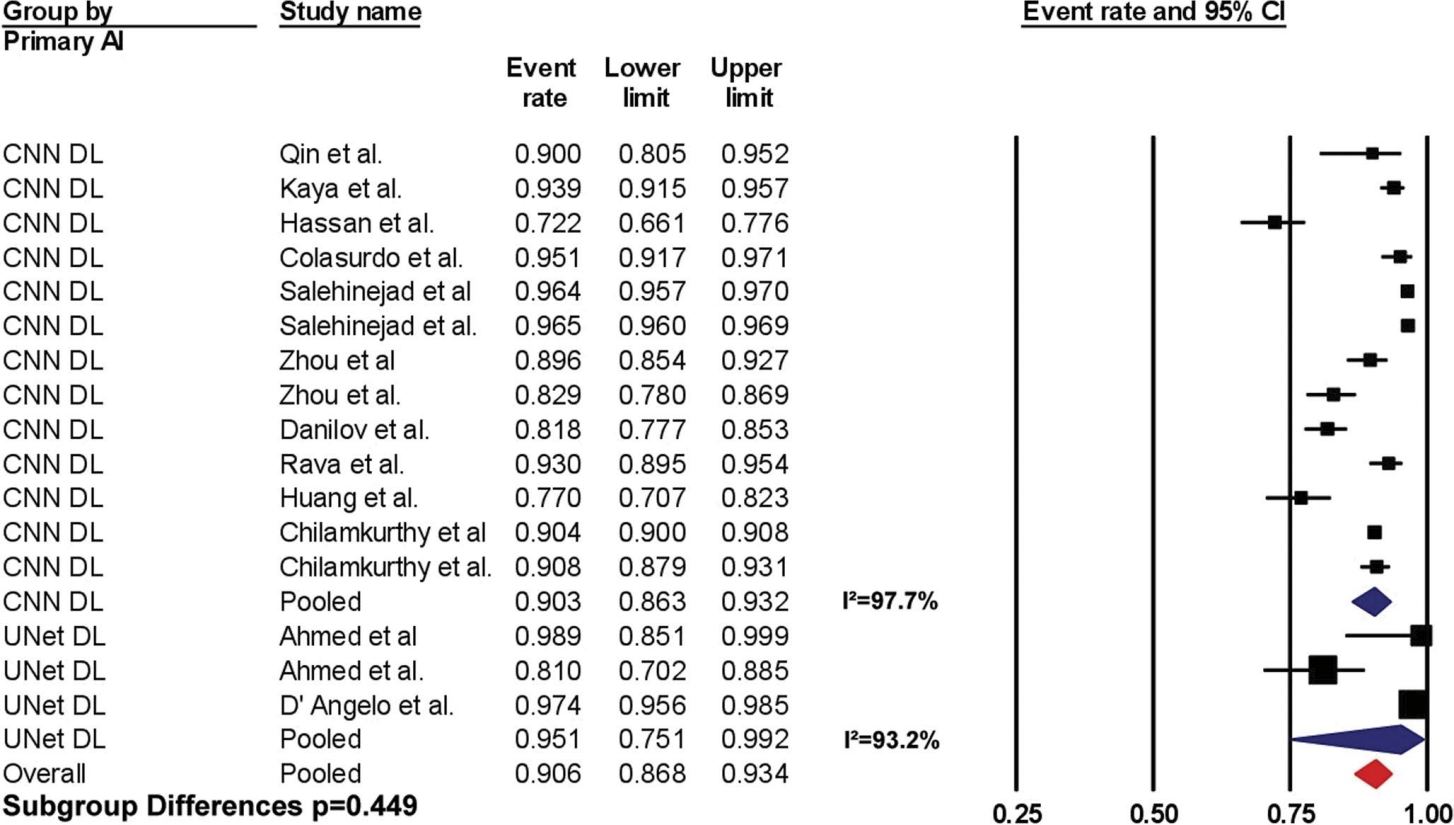
Forest plot depicting pooled accuracy values for CNN DL and U-Net DL architectures. Legend: AI, artificial intelligence; CI, confidence interval; DL, deep learning; CNN, convolutional neural networks

This outcome was reported by 12 studies, which yielded 16 point estimates overall. Among DL models, the pooled accuracy of CNN and U-Net models was 0.903 (95% CI [0.863, 0.932]; I^2^ = 97.7%; *n* = 13) and 0.951 (95% CI [0.751, 0.992]; I^2^ = 93.2%; *n* = 3), respectively. No significant subgroup differences were observed (*p* = 0.449) (Fig. 4). There was no reduction in heterogeneity or change in results on performing sensitivity analysis.

None of the five assessed covariates was significantly associated with the logit event rate in the random-effects meta-regression models (Supplementary Fig. 6A). No significant publication bias was observed (Egger’s intercept: −0.118; *p* = 0.955) (Supplementary Fig. 6B). However, after removing the results from the internal testing dataset evaluated by Chilamkurthy et al., larger sample sizes emerged as a significant predictor of high accuracy (Q = 5.74, df = 1, β = 0.0003, *p* = 0.0167, residual I^2^ = 93.3%) (Supplementary Fig. 6C) [33].

E) Precision

**Fig. 5:**
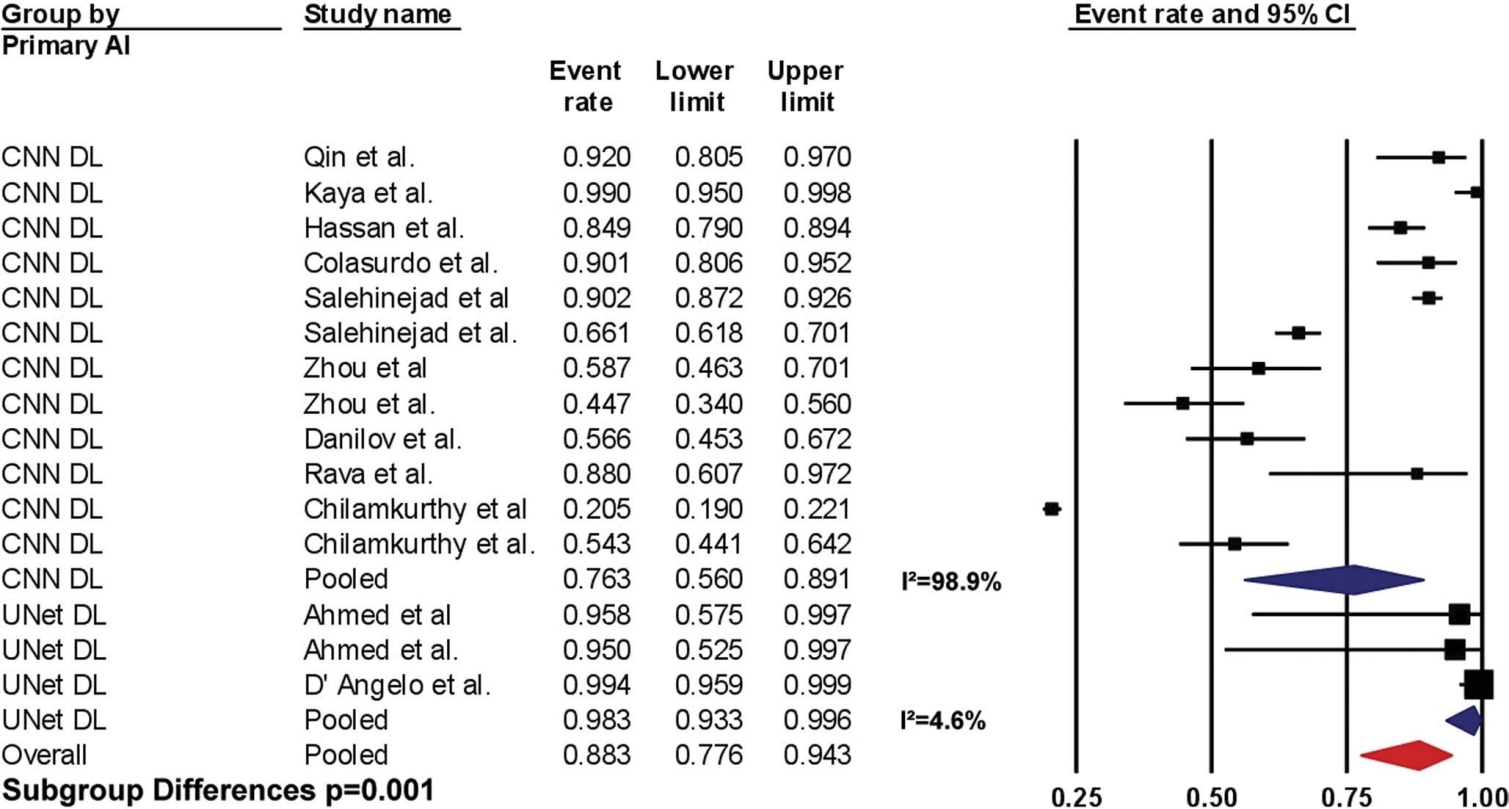
Forest plot depicting pooled precision values for CNN DL and U-Net DL architectures. Legend: AI, artificial intelligence; CI, confidence interval; DL, deep learning; CNN, convolutional neural networks

This outcome was reported by 11 studies, generating 15 point estimates overall. Among DL models, the pooled precision of CNN and U-Net models was 0.763 (95% CI [0.560, 0.891]; I^2^ = 98.9%; *n* = 12) and 0.983 (95% CI [0.933, 0.996]; I^2^ = 4.6%; *n* = 3), respectively (Fig. 5). Significant subgroup differences were observed (*p* = 0.001). There was no reduction in heterogeneity or change in results on performing sensitivity analysis.

The overall heterogeneity was 98.7%. On performing meta-regression analysis, publication year (Q = 17.97, df = 1, *p* < 0.001, residual I^2^ = 94.39%), DL model used (Q = 4.46, df = 1, *p* = 0.034, residual I^2^ = 98.7%), sample size (Q = 8.60, df = 1, *p* = 0.003, residual I^2^ = 94.25%), and model dimensions (Q = 8.09, df = 3, *p* = 0.044, residual I^2^ = 98.7%) emerged as the significant predictors of precision. Recent publication years (β = 0.559, *p* < 0.001), U-Net architecture (β = 2.69, *p* = 0.035), and 3D models (β = 4.27, *p* = 0.022) were associated with significantly higher precision while larger sample size was associated with lower precision (β = −0.0001, *p* = 0.003). Significant publication bias was observed (Egger’s intercept: 7.75; *p* = 0.006) (Supplementary Fig. 7B). On removing results from the internal testing dataset by Chilamkurthy et al., the association between sample size and precision nullified (β = −0.0001, *p* = 0.570), while publication bias also became insignificant (Egger’s intercept: 2.46; *p* = 0.164) (Supplementary Figs. 7 C and D) [33].

## Discussion

Our meta-analysis showcases performance of ML in SDH segmentation. The U-Net architecture emerged as the DL model with the objectively highest sensitivity and precision, while specificity, DOR, and accuracy remained similar across all designs. Notably, these results persisted after removing small studies from meta-analyses of outcomes with significant publication bias. While these results align with those of previous studies, those meta-analyses pooled all intracranial hemorrhage (ICH) cases overall instead of stratifying by hemorrhage type [35, 36]. Additionally, they meta-analyzed diverse DL architectures without determining whether the architectural design itself contributes to variation in performance [13]. By stratifying models based on their architecture, our meta-analysis supports the idea that different architectures are associated with unique error profiles, which is relevant to which architecture is optimal for various clinical settings.

We found that U-Net models performed numerically better than CNN architectures in foreground metrics, attaining higher sensitivity (0.916) and precision (0.983). This is likely due to U-Net’s encoder-decoder structure recovering spatial resolution lost during repeated downsampling by CNNs, thereby allowing precise SDH localization [37, 38]. U-Net addresses this by introducing skip connections which propagate higher-resolution, enable clear demarcation of boundaries, and improve the accuracy of localization [38]. However, it is crucial to note that our results are based off a small number of U-Net studies (*n* = 4) and hence, warrant future studies in larger populations to affirm our findings.

Furthermore, the U-Net architecture demonstrated higher precision and comparable accuracy as compared to CNN in our analysis. Segmentation tasks face a major challenge of foreground-background class imbalance, with background pixels often outnumbering hematoma pixels by a 9:1 ratio in medical imaging datasets [39]. This imbalance causes CNNs trained with standard cross-entropy loss to overpredict positives, reducing precision. On the other hand, U-Net’s segmentation-specific loss functions address this imbalance directly — Dice and hybrid Dice-cross entropy losses penalize false positives, while focal loss down-weights easy negative examples [40, 41]. It is therefore likely that U-Net’s improved precision enables potentially better control over false-positive predictions through these imbalance-aware training strategies. In addition, owing to this imbalance of background pixels, the accuracy values calculated by CNN are likely inflated and hence comparable to that of U-Net models. However, considering that larger sample sizes predicted higher accuracy, the paucity of adequately powered studies pooling U-Net models, necessitates cautious interpretation. Nonetheless, it supports the conclusion that in highly imbalanced prediction problems, pixel-wise foreground metrics should be primarily relied upon [42].

Furthermore, although the U-Net design achieved near-perfect specificity (0.981) and very high DOR values (532.1), these were similar to those observed with CNN (0.899, 54) and hybrid DL (0.919, 76.6) models, with internal testing being a borderline significant predictor of high specificity. Internal testing was often performed on a held-out portion of the training dataset which presumably comprises of more homogenous background pixels than external testing datasets [30, 33]. Hence, it is plausible that all the different DL architectures detected true negatives accurately, thereby ensuring similar specificity. Furthermore, while sensitivity analysis depicted U-Net as being more specific and possessing a significantly higher DOR, these findings are unreliable and likely represent inflated values owing to only 4 test datasets being pooled for the U-Net design as compared to the 22 pooled for CNN models. Therefore, while U-Net’s spatial detail and resolution may be key to reducing false positive findings, future studies training U-Net models and externally validating their findings are mandatory to establish U-Net’s true superiority in this context [11, 43, 44]. Regardless, the overall high DOR values observed across all DL designs suggest the utility of DL models in being particularly useful for accurately distinguishing patients with SDH from those without – a finding that may importantly translate into automating the triage process in the emergency department, thus enabling more efficient workflow and potentially better prognostic outcomes.

Meanwhile, meta-regression revealed that recent publication years and 3D models (compared to 2D models) were significant moderators of high precision values. In this context, all studies utilizing the U-Net architecture were published between 2024 and 25, as compared to those using CNNs, which were published between 2018 and 25; this, thereby suggests that technological advancement over the years, including enhancements of the U-Net architecture, may also play a key role in the higher precision achieved using the U-Net framework [37]. The adoption of new architectures like nnU-Net, which automatically adapts preprocessing steps and network configurations to dataset characteristics, has helped reduce false-positive values in this respect [45]. Meanwhile, 3D models allow for better depth assessment that enables more precise delineation of thinner hematoma collections along the skull, thereby reducing false positives and increasing true negatives. These findings align with those of a multicenter study that reported higher Dice scores using 3D models for auto-segmentation of brain structures as compared to 2.5D and 2D ones [46]. Lastly, while larger sample size seemed to predict lower precision, this was primarily due to the influence of the large internal dataset of one study which tested its model on a retrospective sample of patients with a notably low prevalence of SDH (3%) in comparison to other studies testing their models on datasets enriched with hematoma cases **(**Table 1**)**. Therefore, this study is not truly representative of the trend observed otherwise (Supplementary Fig. 7C) [33].

Overall, the robust diagnostic accuracy demonstrated across all tested DL models positions them as efficient tools for enhancing clinical decision making, ultimately supporting improved patient outcomes. However, AI systems for SDH segmentation should be considered an assistive tool. Studies demonstrate that AI hemorrhage detection can be integrated into emergent CT interpretation workflow in order to support radiologists but does not eliminate the need for radiologists to oversee the workflow [31]. Future research should employ external validation and designs that assess the impact of AI segmentation on clinical workflow, rather than just segmentation accuracy [47].

### Heterogeneity

On performing sensitivity analysis, eliminating the study by Ye et al. reduced heterogeneity of the pooled sensitivity in the hybrid DL subgroup, possibly because this study evaluated a 3D CNN and recurrent neural network (RNN) model as compared to the other models in that subgroup which constituted 2D CNN + RNN algorithms [29]. However, barring this one instance, our results had significantly high heterogeneity values that remained inexplicable by both sensitivity and univariate meta-regression analyses. Given that SDH at different stages presents in variable ways, the stage of SDH, although not specified/reported by most studies, can be a potential source of this heterogeneity [48–51]. Additionally, such inter-study heterogeneity may also be enhanced by the inherent variations across included studies, such as, the type of training dataset, the range of different testing datasets, and the varying ways the models were constructed in each study. Lastly, all meta-analyses conducted were statistically underpowered for performing multivariable meta-regression analyses, since a minimum of ten studies per covariate is required for building a meta-regression model [52]. This may likely account for the high residual heterogeneity observed on univariable meta-regression analyses and hence limits the interpretability of pooled estimates, calling for readers to interpret our findings with considerable caution.

### Limitations and future directions

Our results exhibited high unexplained heterogeneity values as explained above. Additionally, while the U-Net architecture depicted superiority over other DL models, it must be noted that most studies were single centered, with only 4 testing U-Net models compared to the 22 testing CNN architectures. Given this, U-Net models and their multiple variants should be explored further. Furthermore, we were unable to report pooled Dice and Intersection over Union (IoU) values due to inadequate and insufficient reporting. We were also unable to account for the stage of SDH as a potential covariate in meta-regression analyses since only a limited number of studies reported it [31]. Additionally, given the lack of studies comparing ML models and radiologists’ evaluation, it remains unclear whether U-Net models can independently detect SDH or if they should be used as an adjunct to manual segmentation.

In a nutshell, future research should prioritize large, multicenter datasets, standardized outcome measurements, external validation, architecture-specific analyses, analyses specific to hemorrhage type and stage, and direct comparisons with radiologist performance. In addition, patient specific risk factors for SDH development, such as age and anticoagulant usage may also affect SDH’s radiological appearance and therefore, should be accounted for when determining how well ML models segment SDH. Addressing these gaps will be essential for translating AI-based SDH segmentation into the clinical setting.

## Conclusions

This single arm meta-analysis comparing different DL frameworks depicted potential superiority of U-Net models with respect to sensitivity and precision, while specificity, DOR, and accuracy remained consistently high across all DL designs. However, our data for U-Net models was insufficiently powered and mandates future studies in larger cohorts to establish a fair comparison of U-Net and CNN architectures.

## Supplementary Information

Below is the link to the electronic supplementary material.

Supplementary Material 1 (DOCX 348 KB)

## Data Availability

All data was sourced from already published articles, and as such, is readily available online.
