# Supplementary material for: The use of machine learning models for subdural hematoma detection: a single-arm meta-analysis
Source: Neurosurg Rev. 2026 Aug 6;49(1):514. doi: 10.1007/s10143-026-04422-7 (PMC13442323; doi:10.1007/s10143-026-04422-7)
Supplement: Supplementary file 1 [file 10143_2026_4422_MOESM1_ESM.docx]

**Supplementary Material**

**Title: The Use of Machine Learning Models for Subdural Hematoma Detection: A Single-arm Meta-analysis**

**Journal:** Neurosurgical Review

Suraj R. Dumasia^1^, Warda Ahmed^1,2^, Michelle Edavettal^1^, Mackenzie Castellanos^1^, Om H. Gandhi^1^, Giuseppe Lanzino^3^, Omar Choudhri^4*^

**Affiliations:**

^1^Department of Neurosurgery, University of Pennsylvania, Philadelphia, PA

^2^Aga Khan University Medical College, Karachi, Pakistan

^3^Department of Neurosurgery, Mayo Clinic, Rochester, MN

^4^Department of Radiology, University of Pennsylvania, Philadelphia, PA

***Corresponding Author:**

Omar Choudhri, MD

Full Address: 15th Floor South Pavilion, Room 15-146

3400 Civic Center Boulevard

Philadelphia, PA 19104.

Telephone number: 215-349-5548

ORCID: 0000-0002-8311-2336

Email address: [omar.choudhri@pennmedicine.upenn.edu](mailto:omar.choudhri@pennmedicine.upenn.edu) ; [ochoudhri@gmail.com](mailto:ochoudhri@gmail.com)

**Supplementary Table 1: Complete Search Strings**

| **Database** | **Search String** | **Records Identified** |
| --- | --- | --- |
| **PubMed (Medline)** | (((ai artificial intelligence[MeSH Terms]) OR (active machine learning[MeSH Terms]) OR (algorithm, machine learning[MeSH Terms]) OR (deep learning[MeSH Terms]) OR (computational neural network[MeSH Terms]) OR (artificial intelligence) OR (machine learn*) OR (neural network) OR (deep learn*) OR (support vector*) OR (computational intelligence)) AND ((subdural hematoma) OR (SDH) OR (subdural hemorrhage) OR (acute subdural hematoma[MeSH Terms]) OR (chronic subdural hematoma[MeSH Terms]))) | 219 |
| **Cochrane CENTRAL** | (((artificial intelligence[MeSH Terms]) OR (generative artificial intelligence[MeSH Terms]) OR (machine learning[MeSH Terms]) OR (neural networks, computer[MeSH Terms]) OR (support vector machine[MeSH Terms])) OR (machine learn*) OR (neural network) OR (deep learn*) OR (support vector*)) AND ((hematoma, subdural[MeSH Terms]) OR (subdural hematoma*))) | 11 |
| **Scopus** | ((("artificial intelligence") OR ("active machine learning") OR ("machine learning algorithm") OR ("deep learning") OR ("computational neural network") OR ("artificial intelligence") OR (machine learn*) OR ("neural network") OR (deep learn*) OR (support vector*) OR ("computational intelligence")) AND (("subdural hematoma") OR (SDH) OR ("subdural hemorrhage") OR ("acute subdural hematoma") OR ("chronic subdural hematoma"))) | 357 |
| **Embase** | ((('artificial intelligence') OR ('active machine learning') OR ('machine learning algorithm') OR ('deep learning') OR ('computational neural network') OR (machine learn*) OR ('neural network') OR (deep learn*) OR (support vector*) OR ('computational intelligence')) AND (('subdural hematoma') OR (SDH) OR ('subdural hemorrhage') OR ('acute subdural hematoma') OR ('chronic subdural hematoma'))) | 419 |

**Supplementary Table 2: Outcome Definitions**

| **Outcomes** | **Formula used to calculate** |
| --- | --- |
| Sensitivity | $\frac{TP}{TP+FN}$ |
| Specificity | $\frac{TN}{TN+FP}$ |
| Diagnostic Odds Ratio  SE (lnDOR) | $\frac{TP*TN}{FP*FN}$  $\surd(\frac{1}{TP}+\frac{1}{FP}+\frac{1}{FN}+\frac{1}{TN} )$ |
| Accuracy | $\frac{TP+TN}{TP+TN+FP+FN}$ |
| Precision | $\frac{TP}{TP+FP}$ |

TP, true positives; TN, true negatives; FP, false positives; FN, false negatives.

**Supplementary Figure 1: PRISMA Flowchart**

**Records screened by title and abstract:** n= 332

**Records excluded:**

n=254

**Full text articles assessed for eligibility:** n=78

**Records identified through database search:**

PubMed: n=219

Cochrane CENTRAL: n=11

Scopus: n=357

Embase: n=419

**Total: n=1006**

**Total: n= 225**

**Duplicate records** **excluded**:

n=674

**Screening**

**Full text articles excluded: n=56**

Reasons for exclusion

Data could not be extracted (n=29)

Did not report relevant outcomes (n=27)

**Studies included in qualitative synthesis:** n = 22

**Identification**

**Included**

**Studies included in quantitative synthesis:** n = 22

**Supplementary Figure 2: QUADAS-2 Risk of Bias Analysis**

**A)**

Patient Selection

Index Test

Patient Selection

Reference Standard

Flow and Timing

Index Test

Reference Standard

**Risk of Bias**

**Applicability**

**Concerns**

**B)**

**Risk of Bias**

**Applicability**

**Concerns**

**Supplementary Figure 3: Sensitivity Meta-regression and Funnel Plot**

 **A)**

**B)**

**Supplementary Figure 4: Specificity Meta-regression and Funnel Plot**

**A)**

**B)**

**Supplementary Figure 5: Diagnostic Odds Ratio Meta-regression and Funnel Plot**

**A)**

**B)**

**Supplementary Figure 6: Accuracy Meta-regression and Funnel Plot**

**A)**

**B)**

**C)**

**Supplementary Figure 7: Precision Meta-regression and Funnel Plot**

**A)**

**B)**

**C)**

**D)**
